# Identification of Renal Transplantation Rejection Biomarkers in Blood Using the Systems Biology Approach

**DOI:** 10.52547/ibj.3871

**Published:** 2023-08-23

**Authors:** Fatemeh Saberi, Zeinab Dehghan, Effat Noori, Hakimeh Zali

**Affiliations:** 1 Student Research Committee, Department of Medical Biotechnology, School of Advanced Technologies in Medicine, Shahid Beheshti University of Medical Sciences, Tehran, Iran;; 2 Cellular and Molecular Biology Research Center, Shahid Beheshti University of Medical Sciences, Tehran, Iran;; 3 Department of Comparative Biomedical Sciences, School of Advanced Medical Sciences and Technologies, Shiraz University of Medical Sciences, Shiraz, Iran;; 4 Department of Tissue Engineering and Applied Cell Sciences, School of Advanced Technologies in Medicine, Shahid Beheshti University of Medical Sciences, Tehran, Iran

**Keywords:** Biomarkers, Gene regulatory network, Renal diseases

## Abstract

**Background:**

Renal transplantation plays an essential role in the quality of life of patients with end-stage renal disease. At least 12% of the renal patients receiving transplantations show graft rejection. One of the methods used to diagnose renal transplantation rejection is renal allograft biopsy. This procedure is associated with some risks such as bleeding and arteriovenous fistula formation. In this study, we applied a bioinformatics approach to identify serum markers for graft rejection in patients receiving a renal transplantation.

**Methods:**

Transcriptomic data were first retrieved from the blood of renal transplantation rejection patients using the GEO database. The data were then used to construct the protein-protein interaction and gene regulatory networks using Cytoscape software. Next, network analysis was performed to identify hub-bottlenecks, and key blood markers involved in renal graft rejection. Lastly, the gene ontology and functional pathways related to hub-bottlenecks were detected using PANTHER and DAVID servers.

**Results:**

In PPIN and GRN,* SYNCRIP, SQSTM1, GRAMD1A, FAM104A, ND2, TPGS2, ZNF652, RORA*, and *MALAT1 *were the identified critical genes. In GRN, *miR-155, miR17, miR146b, miR-200* family, and *GATA2 *were the factors that regulated critical genes. The MAPK, neurotrophin, and TNF signaling pathways, IL-17, and human cytomegalovirus infection, human papillomavirus infection, and shigellosis were identified as significant pathways involved in graft rejection.

**Conclusion:**

The above-mentioned genes can be used as diagnostic and therapeutic serum markers of transplantation rejection in renal patients. The newly predicted biomarkers and pathways require further studies.

## INTRODUCTION

Renal transplantation significantly improves the quality of life of patients with ESRD and increases their survival rate. It is recognized as the most effective treatment for patients with chronic renal failure^[^^1^^]^. One of the leading causes of allograft dysfunction in renal transplantation is immune-suppressive therapy. In recent years, surgical methods and immunosuppressive drugs have made impressive progress; however, the results of renal transplantation have remained unacceptable^[^^2^^]^ and have not been improved substantially over the years^[^^3^^]^.

Three main types of allograft rejection after renal transplantation include hyper-acute, acute, and chronic allograft rejections^[^^4^^]^. Renal function within the first year of transplantation is an essential factor influencing the graft survival^[^^5^^]^. Acute rejection increases the risk factors for short- and long-term allograft survival^[^^6^^]^; up to 12% of patients on renal transplantation waiting list are re-transplanted^[^^3^^]^. Studies have shown that the gene expression profiles of rejecting and non-rejecting renal tissues and serum are different^[^^7^^,^^8^^]^. Genetic variability may explain the different mechanisms of renal transplantation rejection^[^^9^^,^^10^^]^. In addition to genetic factors, environmental factors, including ischemia-reperfusion injury and degree of immunosuppression, have a link with acute rejection^[^^8^^]^.

The renal allograft biopsy is an invasive procedure associated with some risks such as bleeding and arteriovenous fistula formation. In addition, it has biopsy-associated costs and inter-observer variability in biopsy specimen scoring. Therefore, identification of genetic biomarkers will help preventing the occurrence of these issues^[^^5^^,^^6^^]^. 

Rapid advancements in various technologies, including genomics, transcriptomics, proteomics, microbiomics, and metabolomics, have promoted a better understanding of graft injury mechanisms and created new development in medical science. The systems biology approach integrates the extensive generated omics data for a deeper understanding of the pathophysiology of renal allograft rejection. A large amount of omics data obtained using various technologies such as transcriptomic, proteomics, and metabolomics provide more accurate diagnosis and highly individualized treatment^[^^2^^]^. The study design with larger sample sizes and analysis of genomic relationships using bioinformatics tools is vital in determination of biomarkers^[^^8^^]^.

Despite various studies on renal transplantation rejection in the field of genetics, there are no definitively identified genetic predictors for renal allograft rejection. Furthermore, the biological functions of the identified genes have not been determined, and, hence, the results are mostly unreliable. On the other hand, molecular mechanisms involving the rejection cause of renal transplantations are still not comprehensively understood. In this study, we first used the renal transplantation PPIN and GRNs to identify important genes and their molecular mechanisms contributed to the transplantation rejection. Indeed, identification of the critical genes in the blood can be a non-invasive method for understanding renal transplantation rejection in the early stages. 

## MATERIALS AND METHODS

**Study design**

This study was conducted in four main steps: (1) selection of appropriate GSE regarding microarray data from healthy blood samples and renal transplantation patients, (2) identification of DEGs in datasets and selection of the shared DEGs among datasets, (3) construction of a PPIN and GRN from the shared DEGs, and (4) functional enrichment analysis of the target genes. The graphical workflow of this study is represented in Supplementary Figure 1. 

**Data collection**

The microarray datasets of blood samples from renal transplantation patients and healthy individuals were retrieved from the GEO database (www.ncbi.nlm.nih. gov/geo). Two datasets, GSE15296 and GSE46474 in the Platform Affymetrix Human Genome U133 Plus 2.0 Array (GPL570), were chosen for further analysis in this study.

**Raw data analysis**

The online GEO engine, GEO2R (https://www.ncbi. nlm.nih.gov/geo/geo2r/), together with affylmGUI, an R-based package, was utilized for data normalization. The DEGs were filtered based on log2FC > 0.5 or < -0.5 fold and a p value < 0.05. Finally, the shared DEGs were selected from GSE15296 and GSE46474 using a Venn diagram for future analysis. 

**Construction of PPIN**

The shared DEGs from the two GSE datasets were fed into the HIPPIE database (http://cbdm-01.zdv.uni-mainz.de/~mschaefer/hippie/) for interaction network assembly. The connection nodes with a confidence score threshold 0.7 were loaded into Cytoscape v3.5.1 software. 

**Clustering and topological analysis of the PPIN**

The MCODE app and Network Analyzer (plugged in Cytoscape) were used to identify sub-networks and analyze the topological parameters, including degree (hub), betweenness centrality (bottleneck), and closeness centrality, respectively. The highly connected proteins were considered hubs^[^^11^^,^^12^^]^. The network nodes with several shortest paths were defined as bottlenecks^[^^13^^]^, and the closeness centrality was found to be a key contributor to central network nodes in PPIN^[^^14^^]^. The shared nodes between 10% degree, 10% betweenness centrality, and 10% closeness centrality were chosen using a Venn diagram. These nodes were used for subsequent analyses. The MCODE app identified sub-networks based on degree threshold = 2, node score threshold = 0.2, k-core = 2, and maximum depth of 100^[^^15^^,^^16^^]^. 

**Analysis of GRN**

Five relationships, miR-gene, miR-TF, TF-gene, TF-miR, and lncRNA-gene, were identified to construct two GRNs for the shared up- and down-regulated DEGs, separately. 

**Identification of TFs regulating genes**

The databases TRANSFAC (http://genexplain.com/ transfac/) and TRRUST v2 (https://www.grnpedia.org/ trrust/) were employed to find the TFs regulating DEGs^[^^17^^]^. The TRRUST v2 is a curated database of 6552 TF-target interactions for 828 TFs in mouse and 8444 regulatory interactions for 800 TFs in humans^[^^18^^]^.

**Identification of miRNAs suppressing genes and TFs **MiRNAs regulating our DEGs and TFs were extracted from the miRTarBase release 8.0 (http://mirtarbase. mbc.nctu.edu.tw/) and miRecords (http://c1.accura science.com/miRecords/). The miRTarBase database has more than 13,404 experimentally validated miRNA-target gene interactions^[^^19^^]^. The miRecords is a helpful resource for validated miRNA-gene interactions in seven animal species^[^^20^^]^. 

**Identification of TFs regulating miRNAs**

Curated TF-miRNA regulations were extracted from the TransmiR v2.0 (http://www.cuilab.cn/transmir) database, an open-source database of 623 TFs and 785 miRNAs for 19 organisms^[^^21^^]^. 

**Identification of lncRNAs regulating DEGs**

LncRNAs that regulated our DEGs were collected from the LncRNA2Target v2.0 (http://123.59.132.21/ lncrna2target) database, which is a comprehensive resource of lncRNA-gene interactions^[^^22^^]^. 

**Construction of GRN**

Five relationships, miR-gene, miR-TF, TF-gene, TF-miR, and lncRNA-gene, were merged in Cytoscape software for up- and downregulated genes, separately. Five percentage of nodes with the highest degree, betweenness centrality, and closeness centrality were identified using the Network Analyzer tool in the up- and downregulated gene networks. Finally, shared nodes with the highest degree, betweenness centrality, and closeness centrality were selected using a Venn diagram for future analysis. 

**Functional enrichment analysis**

The top genes (hub-bottleneck) from PPIN, GRN, and sub-network were selected for gene ontology (biological process, molecular function, and cellular component) and KEGG pathway analyses. These genes were studied using PANTHER (http://www.pantherdb.org/) and DAVID (https://david.ncifcrf.gov) tools for gene ontology and the KEGG pathway analyses, respectively.

## RESULTS

**Raw data analysis and identification of DEGs**

From datasets GSE15296 and GSE46474, 95 shared DEGs, including 39 upregulated and 56 downregulated genes were identified. The results of the Venn diagram are represented in Supplementary Table 1. 

**Construction of PPIN, clustering, and topological analysis**

**Fig. 1:**
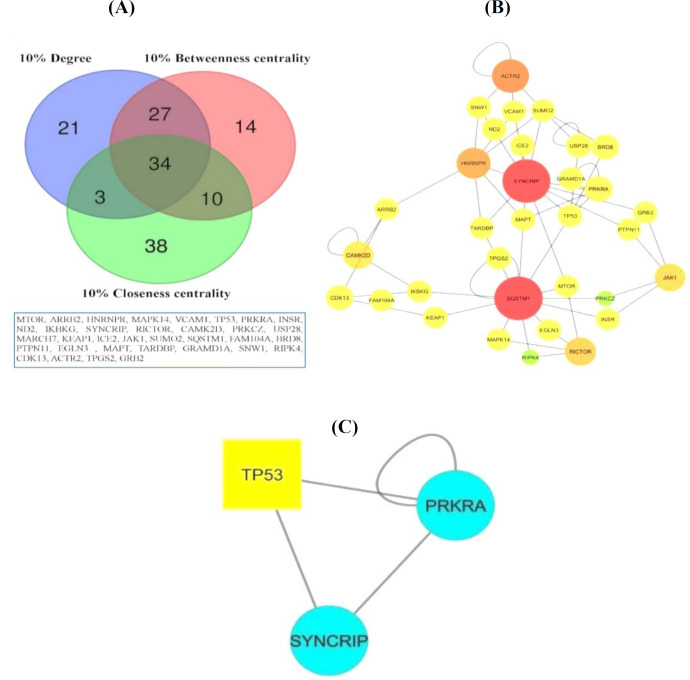
PPIN. (A) The results of shared nodes between highest degree, betweenness centrality, and closeness centrality in PPIN obtained by Venn diagram; (B) PPIN of shared nodes. Nodes with a red color and bigger sizes have the highest degree, and nodes with a green color and smaller sizes have the lowest degree; (C) the sub-network obtained using the MCODE app

PPIN for 95 shared DEGs was constructed using the HIPPIE database. We used Cytoscape software 3.5.1 to visualize the PPIN, as well as MCODE app and Network Analyzer tools to identify sub-networks and topological network properties, hub-bottleneck nodes. The topological network properties included a network density of 0.003, the shortest path of 93%, average number of neighbors of 2.247, and diameter of 12. The 34 shared nodes with the highest degree, betweenness centrality, and closeness centrality, their PPIN, and MCODE cluster are shown in Figure 1. The *SYNCRIP* and *SQSTM1* genes were identified as the highest degree nodes, and the *GRAMD1A*,* FAM104A*,* ND2*, and *TPGS2* genes were selected as the highest betweenness centrality and closeness centrality. Table 1 shows genes and topological properties of degree, betweenness centrality, and closeness centrality. A list of 10% of the genes with the highest degree, betweenness centrality, and closeness centrality is available in Supplementary Table 2. The MCODE app was used to find highly interconnected regions in PPIN. MCODE app results showed one sub-network with score = 3, nodes = 3, and edge = 4. 

**Identification of miRNA-gene/TFs, TF-miRNA/ gene, and lncRNA-gene interactions**

The upregulated genes were targeted with 431 miRNAs obtained from miRTarBase and miRecords through 575 interactions. Also, 153 TFs regulated target genes with 569 interactions; these TFs were identified 

using TRRUST and TRANSFACT. In addition, 1441 miRNAs suppressed 133 TFs with 5636 interactions. The TransmiR database is used to identify TFs regulating miRNAs. This database has 353 TFs regulated 320 miRNAs with 2026 interactions. The lncRNAs regulating target genes were identified by LncRNA2Target v2. In this regard, 2899 lncRNA regulated the target genes with 3011 interactions. The downregulated genes were regulated with 823 miRNAs, 183 TFs, and 73 lncRNAs through 1542, 897, and 220 interactions, respectively. Overall, 355 TFs were detected to regulate 325 miRNAs with 2025 interactions, and 1488 miRNAs suppressed 164 TFs through 6654 interactions. These results are represented in Table 2.

**Construction of GRN**

**Fig. 2:**
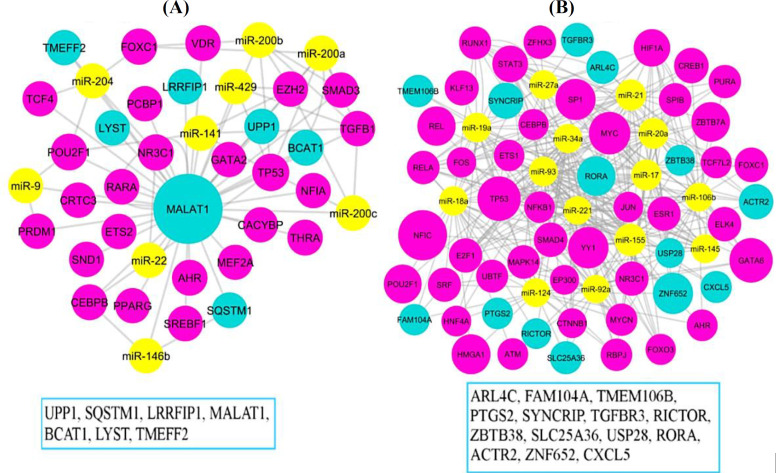
GRNs. (A) Upregulated and (B) downregulated genes, sub-networks of shared nodes between the highest degree, betweenness centrality, and closeness centrality. Nodes with bigger sizes have the highest degree. The miRNAs, TFs, and genes are shown with yellow, pink, and blue colors, respectively

The relationships between miR-gene, miR-TF, TF-miR, TF-gene, and lncRNA-gene were separately merged and visualized in Cytoscape software for up- and downregulated genes. Five percent of the nodes with the highest degree, betweenness centrality, and closeness centrality were identified with the Network Analyzer, and shared nodes were detected using a Venn diagram (Supplementary Table 3 and Supplementary Table 4). In the upregulated gene network, *MALAT1* lncRNA is considered a hub where the regulation of most genes is upregulated in the network. Various regulation factors of miRNAs (*miR-200a*, *miR-200b*,* miR-200c*,* miR-146b*,* miR-429*,* miR-204*,* miR-141*,* miR-22*, and *miR-9*) and several TFs were associated with *MALAT1* gene. On the other hand, the *ZNF652* and *RORA* genes are considered as hubs in the downregulated gene network. The *ZNF652* gene was regulated by miRNAs (*miR-106b*, *miR-17*,* miR-93*, *miR-20a*, and *miR-155*) and TFs (*FOXC1*,* HNF4A*, *MYCN*,* RUNX1*,* SRF*, and *YY1*). The same miRNAs, as well as TFs (*CBEPB*,* E2F1*,* ESR1*,* ETS1*, *GATA6*, *HIF1A*, *HNF4A*, *KLF13*,* NFIC*,* NFKB1*, *RUNX1*,* SP1*, and *ZFHX3*) were regulated by *RORA* gene expression. Figure 2 shows the shared nodes in the up- and downregulated gene network.

**Table 1:** List of 10% shared nodes with the highest degree, betweenness centrality, andcloseness centrality in PPIN

**Gene names**	**Degree**	**Betweenness centrality**	**Closeness centrality**
*SYNCRIP*	126	0.391581	0.303704
*SQSTM1*	104	0.323407	0.2914
*ACTR2*	63	0.161765	0.241035
*JAK1*	48	0.143194	0.254658
*RICTOR*	41	0.150494	0.262484
*CDK13*	28	0.077687	0.249089
*HNRNPR*	26	0.083022	0.278344
*BRD8*	18	0.047761	0.240964
*PRKRA*	16	0.029893	0.256731
*CAMK2D*	14	0.047703	0.244922
*MARCH7*	11	0.024044	0.235835
*USP28*	9	0.020345	0.238372
*SUMO2*	6	0.085407	0.266234
*GRAMD1A*	5	1	1
*TP53*	4	0.02893	0.26098
*SNW1*	4	0.04649	0.260152
*IKBKG*	4	0.043812	0.247959
*ARRB2*	4	0.028103	0.242317
*FAM104A*	3	1	1
*ND2*	3	1	1
*TPGS2*	3	1	1
*MTOR*	3	0.076921	0.286313
*TARDBP*	3	0.034267	0.27628
*MAPT*	3	0.03553	0.274615
*PTPN11*	3	0.023117	0.253165
*GRB2*	3	0.022892	0.253008
*VCAM1*	3	0.019787	0.252153
*KEAP1*	3	0.029272	0.249164
*MAPK14*	3	0.040559	0.247809
*INSR*	3	0.023868	0.242963
*ICE2*	3	0.036708	0.242388
*RIPK4*	3	0.015006	0.241817
*EGLN3*	3	0.016639	0.239556
*PRKCZ*	2	0.01777	0.242245

**Table 2:** Summary of five regulatory relationships among miRNA-gene, TF-Gene, miR-TF, TF-miR, and lncRNA-gene interactions

**Relationship**	**No. of pairs**	**No. of genes**	**No. of TFs**	**No. of miRNAs**	**No. of lncRNAs**
Upregulated					
miR-gene	575	29	-	431	-
miR-TF	5636	-	133	1441	-
TF-Gene	569	31	153	-	-
TF-miR	2026	-	353	320	-
lncRNA-gene	3011	31	-	-	2899
					
Downregulated					
miR-gene	1542	50	-	823	-
miR-TF	6654	-	164	1488	-
TF-Gene	897	51	183	-	-
TF-miR	2025	-	355	325	-
lncRNA-gene	220	32	-	-	73

**Functional analysis**

**Fig. 3:**
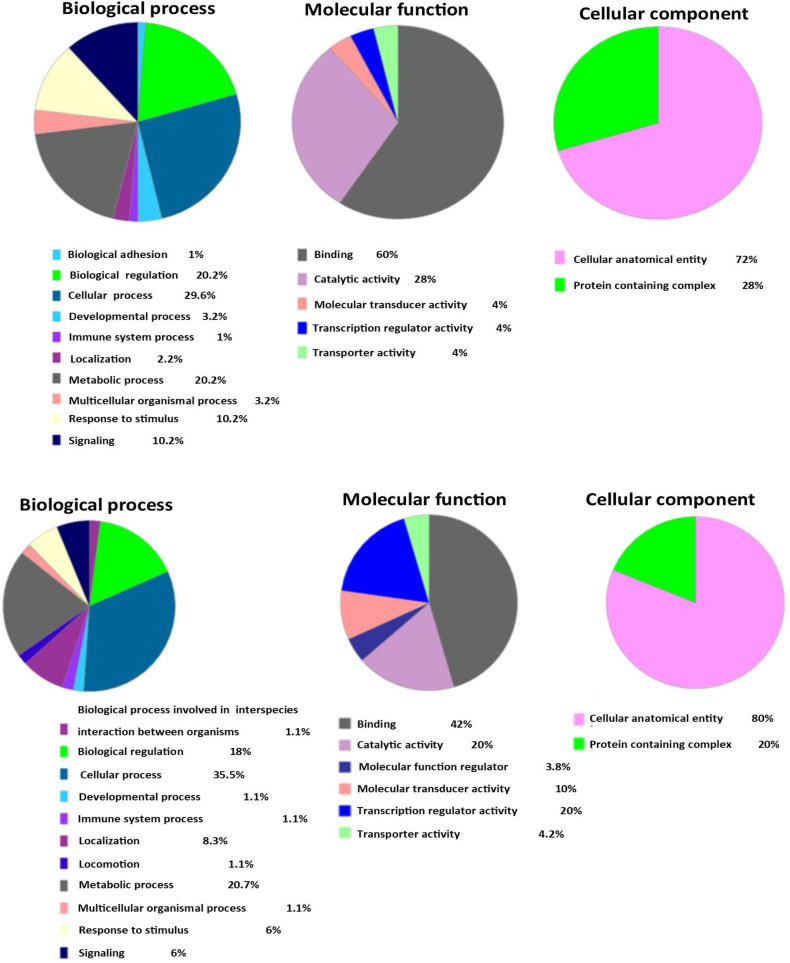
Top gene ontology for shared nodes between the highest degree, betweenness centrality, and closeness centrality in PPIN/sub-network (A) and GRN (B) using the PANTHER tool, respectively

For gene ontology, the shared genes from the highest degree, betweenness centrality, and closeness centrality in PPIN, GRN, and sub-network were submitted to the PANTHER tool. In PPIN, the biological adhesion, developmental process, immune system process, metabolic process, response to stimulus, and signaling were the top biological processes. In molecular function terms, binding catalytic activity, molecular transducer, transcription regulator, and transporter activity were remarkable. The most nodes shared in PPIN were present in the cellular anatomical entity and protein-complex (Fig. 3A). The top biological process terms for 21 shared nodes were obtained from 5% of the highest degree, betweenness centrality, and closeness centrality in GRN (up- and downregulated), including the biological process involved in interspecies interaction between organisms, biological regulation, develop-mental process, immune system process, locomotion, metabolic process, response to stimulus, and signaling. Significant molecular function terms included binding, catalytic activity, molecular function regulator, molecular transducer activity, transcription regulator activity, and transporter activity. In cellular component term, the cellular anatomical entity and protein complex were significant. These results are represented in Figure 3B. We performed KEGG pathway analysis using the DAVID database for the shared node genes from the highest degree, betweenness centrality, and closeness centrality in PPIN and GRN. The top pathways included the MAPK signaling pathway, human cytomegalovirus infection, neuro-trophin signaling pathway, shigellosis, and human papillomavirus infection in PPIN. In GRN, the IL-17 signaling and TNF signaling pathways were significant pathways (Table 3). 

**Table 3:** Result of top KEGG pathways available in PPIN, GRN, and sub-network

**ID**	**Term**	**FDR**	**Genes**
PPIN and sub-network			
hsa04010	MAPK signaling pathway	0.002078	*INSR, GRB2*,* IKBKG*,* ARRB2*,* MAPT*,* MAPK14*,* TP53*
hsa05163	Human cytomegalovirus infection	0.002778	*GRB2*,* IKBKG*,* MAPK14*, *TP53*,* MTOR*,* JAK1*
hsa04722	Neurotrophin signaling pathway	0.002778	*CAMK2D*,* GRB2*, *PTPN11*,* MAPK14*,* TP53*
hsa05131	Shigellosis	0.003221	*ACTR2*,* IKBKG*,* MAPK14*,* TP53*,* SQSTM1*,* MTOR*
hsa05165	Human papillomavirus infection	0.007904	*GRB2*,* IKBKG*,* TP53*,* PRKCZ*,* MTOR*,* JAK1*
GRN			
hsa04657	IL-17 signaling pathway	1	*PTGS2*,* CXCL5*
hsa04668	TNF signaling pathway	1	*PTGS2*, *CXCL5*

FDR, false discovery rate

## DISCUSSION

Renal transplantation is the most effective treatment option for patients with a final-stage chronic renal disease^[^^23^^]^. The allograft biopsy in patients receiving renal transplantation is an expensive and invasive procedure with inter-observer variability that can cause graft rejection. As a result, investigating genetic biomarkers in blood samples would be promising. In the present study, we used experimental data obtained from the blood of patients receiving renal transplantations to form a public database to suggest effective genetic biomarkers involving in graft rejection.

In this study, we investigated renal transplantation-related DEGs in the blood of two groups; rejected grafts and normal. These DEGs were used to identify critical targets and molecular mechanisms contributing to graft rejection. Our analysis of PPIN demonstrated *SYNCRIP*,* SQSTM1*,* GRAMD1A*,* FAM104A*, *ND2*, and *TPGS2* as critical genes that regulate renal rejection in patients receiving the graft. On the other hand, investigation of GRN showed that *ZNF652*,* RORA*, and *MALAT1* lncRNA were crucial hubs. These genes can suggest biomarker panels and drug targets in the diagnosis and therapy of renal transplantation rejection.

The functional enrichment analysis revealed the critical genes involved in the MAPK signaling pathway, including human cytomegalovirus and papillomavirus infection, shigellosis, and neurotrophin, IL-17, and TNF signaling pathways. In this study, we hypothesized how some of these critical genes and pathways could regulate graft rejection in transplanted patients.

*SYNCRIP* and *SQSTM1* were downregulated hub genes in our PPIN. The knockdown of synaptotagmin-binding cytoplasmic RNA-interacting protein, called *SYNCRIP* (*hn RNA-Q* or *NSAP1*), disrupts miRNA sorting in the exosome. This protein binds to a specific miRNA in enriched exosomes through the hExo motif and participates in miRNA localization and miRNA loading into the exosome^[^^24^^]^. SYNCRIP/U2AF2 interaction plays an essential role in immune pathways, including T cell activation. In 2015, Whisenant et al.^[^^25^^]^ reported that the knockdown of *SYNCRIP* or *ILF2* could reduce the secretion of IL-21, a cytokine for activation of T follicular helper cells, which have a crucial role in graft rejection and chronic inflammatory disorders. SQSTM1 protein is an oncogene that its overexpression in clear cell renal cell carcinoma increases resistance to redox stress, and its reduction has the opposite effect and reduces tumor formation^[^^26^^]^. In 2014, Zotti et al.^[^^27^^]^ reported that the expression of *p62/SQSTM1*, as an autophagic marker, increases in allograft biopsies of transplantation-receiving renal patients suffering from polyomavirus hominis 1 virus nephropathy, which degrades the mitochondria. However, our study showed a decreased level of *SQSTM1* in the tissue of transplanted rejected patients. Therefore, the SQSTM1 mechanism may play a role in graft rejection, which requires further investigation. 

*GRAMD1A* is an upregulated hub gene in our PPIN. *GRAMD1A* is expressed in all types of cancer tissues. This gene is closely related to immune checkpoint genes. The adverse impacts of GRAMD1A in renal clear cell carcinoma largely depend on the immunomodulatory effects of TILs in the tumor microenvironment, which can make it a biomarker for renal diseases^[^^28^^]^. GRAMD1A has a vital role in autophagosome biogenesis through cholesterol distribution^[^^29^^]^. Chen et al.^[^^30^^]^ reported that autophagy could increase graft rejection in transplanted patients. Günther et al.^[^^31^^]^ showed that *GRAMD1A* is overexpressed in renal transplantation rejection cases. Therefore, targeting this gene can prevent renal rejection. Mitochondrial *ND2* and *TPGS2* are other hubs in PPIN that are downregulated in patients with renal graft rejection. One of the essential functions of the *ND2* gene is the regulation of mitochondrial-dependent apoptosis^[^^32^^]^. Roedder et al.^[^^33^^]^ have reported that mitochondria act a crucial role in graft rejection and can be targeted for immunosuppression. In Wang et al.’s^[^^34^^]^ study, circular RNA related to *TPGS2* has been involved in cancer metastasis through changes in tumor microenvironment. Overexpression of *TPGS2* leads to significant cell migration and increased production of pro-inflammatory chemokines. However, in cancer metastasis, it facilitates the *miR-7*/TRAF6/NF-kB signaling cascade, which renal transplantation rejection might follow this pattern. The molecular mechanism of *ND2* and *TPGS2* are still unclear in graft rejection; therefore, further study on these genes may introduce new therapeutic targets.

This study showed that *ZNF652* and *ROR* genes are hub-bottlenecks in GRN that are downregulated in patients with renal graft rejection. ZNF652 is a TF binds to consensus DNA sequence and regulates various cell processes^[^^35^^]^. One of the *ZNF652*-regulated miRNAs is *miR-155*. Yin et al.^[^^36^^]^, using UTR receptor analysis, showed that *miR-155* targets *ZNF652* and modulates gene expression in Epstein-Barr Virus^[^^36^^]^. This virus increases the risk of post-transplantation lympho-proliferative disorder in renal transplantation recipients^[^^37^^]^. Other miRNAs and TFs that regulate *ZNF652* show an unclear function in graft rejection and requires further investigations.

The *ROR* from the subfamily of ROR nuclear receptors mediates essential cellular adaptation to hypoxia, which is a potential endogenous protector in renal ischemic injury. Its activation is a promising therapeutic strategy for preventing acute renal injury. The harmful effects of *ROR* deficiency are attributed to the apoptosis of tubular epithelial cells, resulting in renal inflammation and oxidative stress^[^^38^^]^. Several regulatory factors, including microRNAs and TFs, modulate the expression and function of this gene (Fig. 3). In a study performed by Liu et al.^[^^39^^]^, *miR-17-92*-deficient cells showed an increase in *RORA* expression level. That study also showed *miR-17-92* deficiency, increased *RORA* level, and reduced Th17 differentiation. Mycko et al.^[^^40^^]^ demonstrated the enhancement of Th17 cell differentiation by the upregulation of *miR-155-3p* in CD4^+^ T cells. Th17 cells contribute to acute and chronic allograft injury in renal transplantation recipients. Strategies targeting the Th17 pathway can improve allograft outcomes^[^^41^^]^. Therefore, a reduction in this gene causes severe damage to the kidney.

*MALAT1 *is an lncRNA that is upregulated in the GRN of renal transplantation rejection patients. This lncRNA plays an essential role in various physiological processes, including nuclear organization, epigenetic change in gene expression, and alternative splicing^[^^42^^]^. The *miR-146b *is one of the miRNAs that regulate *MALAT1* expression (Fig. 3). Paterson et al.^[^^43^^]^ reported that *miR-146b-5p* upregulation in rat models causes *chronic kidney disease* (CDK) and severe progression. The *miR-146b-5p* can also increase the expression of *MALAT1*^[^^44^^]^. Non-coding RNA (*MALAT1*) is crucial in the pathophysiology of acute renal injury. Groeneweg et al.^[^^45^^]^ reported *MALAT1* expression as a vascular injury marker in patients with simultaneous pancreas-renal transplantations. Xiong et al.^[^^46^^]^ reported that expression of *miR-200* family, e.g. *miR-200a*, *miR-200b*,* miR-200c*, and *miR-141*, were downregulated in the early phase of unilateral ureteral obstruction and caused chronic renal injuries. The *MALAT1 *lncRNA can sponge *miR-200c* and suppress its function^[^^47^^]^. The GATA2 is one of the TFs that regulate *MALAT1 *expression (Fig. 3). GATA2 increases oxidative stress and upregulates inflammatory cytokines in renal ischemia-reperfusion injury^[^^48^^]^; however, the interaction between GATA2 and *MALTA1* remains unknown. Therefore, *MALAT1* can be introduced as a novel diagnosis and therapeutic target.

The functional pathways regulated by key hubs in PPIN include the MAPK and neurotrophin signaling pathways, human cytomegalovirus and papillomavirus infections, and shigellosis. Mitogen-activated protein kinases regulate cellular processes such as proliferation, differentiation, death, and survival in renal disease. Experimental evidence has shown that MAPK pathway is responsible for the pathogenesis of renal disease^[^^49^^]^. One of the roles of the MAPK pathway is the activation of T cells. Vafadari et al.^[^^50^^]^ reported that the inhibition of p38 MAPK signaling in T cells could decrease transplantation rejection in renal patients. Human cytomegalovirus tends to invade the allograft due to changes in the immune system in the allograft cases and the presence of virus within latent cells of the allografted tissue obtained from the donors. In patients who have undergone renal transplantations, it manifests as nephritis^[^^51^^]^.

NTs or neurotrophic factors are growth factors required for regulation, maintenance, and renewal of certain nerve cells in the brain. BDNF is a group of NTs that act as crucial molecules in neurological diseases. Stimulation of NTs can be associated with the positive regulation of antioxidant systems but prevents the formation of several inflammatory mediators, including NF-κB and TNF-. It also induces the tropomyosin receptor kinase cascade and antiapoptotic effects, as well as produces an antioxidant in neurons^[^^52^^]^. While renal transplantation is a preferred option for ESRD, delayed graft function is a major problem affecting the long-term renal survival. In addition, BDNF plays an essential role in reducing ischemia/reperfusion injury. In Molnar's study^[^^53^^]^, BDNF serum levels were shown to be lower in ESRD patients than that in the healthy individuals. In transplantation recipients, this factor decreases, suggesting BDNF as a new biomarker in graft function after renal transplantation. Lu et al.^[^^54^^]^ have demonstrated that Ospc3 effector protein of the Shigella pathogen, which involves in the ERK and P38 MAPK phosphorylation signaling, contributes to bacterial infection and cell proliferation in the periphery of infected foci, and it is caused by the activation of mTOR signaling^[^^54^^]^. Shigellemia is often observed in patients with renal transplantations who have immune-suppressed immune systems^[^^55^^]^. Studies have suggested an increased risk of anogenital warts in renal transplantation recipients compared to control groups^[^^56^^,^^57^^]^. HPV reactivity elicits an adequate immune response to all four HPV types in transplanted and dialysis patients^[^^58^^]^. Therefore, shigellosis and anogenital warts may affect renal transplantation patients. 

The IL-17 and TNF signaling pathways are functional pathways regulated by a critical hub in GRN. Loss of IFN-γ causes rapid graft rejection and increases parenchymal necrosis in recipients. IFN-γ is a negative regulator of Th17, and its deficiency leads to the increased IL-17 production and neutrophil infiltration^[^^59^^]^. IL-17 is essential for inflammation and inflammatory response and involves in the pathogenesis of allograft transplantation^[^^60^^]^. TNS is a strong cytokine that increases the inflammatory response in renal transplantations^[^^61^^]^. Its role in renal inflammation indicates a complex interaction between immune effector cells and innate renal cells. 

 Our study identified critical genes and molecular mechanisms involving in the pathogenesis of renal transplantation rejection. This study used a network-based approach (PPIN and GRN) to identify critical hubs in the blood, as well as the molecular mechanisms involved in graft rejection in renal transplantation patients. PPIN and GRN hubs and bottlenecks that help as a diagnostic panel for transplant rejection in renal patients include *SYNCRIP, SQSTM1, GRAMD1A, FAM104A, ND2, TPGS2, RORA, ZBTB38* and *MALAT1* genes. The hubs and bottlenecks regulate functional pathways, including MAPK signaling pathway, human cytomegalovirus infection, neurotrophin signaling pathway, shigellosis, human papillomavirus infection, IL-17, and TNF signaling pathways. Since the renal allograft biopsy is an invasive and expensive procedure associated with bleeding and arteriovenous fistula formation, using noninvasive methods such as serum markers to diagnose renal transplantation rejection could reduce possible risks. We hope our analysis will help identify diagnosis and therapeutic graft rejection biomarkers in renal transplantation patients in the early stages of rejection. While some of our results are in consistent with previous studies, some others require further in vitro and in vivo investigation. 

## DECLARATIONS

### Acknowledgments

This study is related to project NO.1399/62245 from the Student Research Committee, Shahid Beheshti University of Medical Sciences, Tehran, Iran. We appreciate the Student Research Committee and Research and Technology Chancellor at Shahid Beheshti University of Medical Sciences, Tehran, Iran for the financial support of this study.

### Ethical statement

This study was approved by the Ethics Committee of Shahid Beheshti University of Medical Siences, Tehran, Iran (ethical code: IR.SBMU.RETECH.REC.1399. 1052).

### Data availability

The data supporting the findings of this study are available from the corresponding author upon reasonable request.

### Author contributions

FS and ZD: performed the analysis and helped in writing of manuscript; EN: helped in writing of manuscript; HZ designed the research study.

### Conflict of interest

None declared.

### Funding/support

This study is supported by Student Research Committee and Research and Technology Chancellor at Shahid Beheshti University of Medical Sciences, Tehran, Iran (grant number: 1399/62245).

## Supplementary Materials


