# Supplementary material for: Identification of Renal Transplantation Rejection Biomarkers in Blood Using the Systems Biology Approach
Source: Iran Biomed J. 2023 Aug 23;27(6):375–87. doi: 10.52547/ibj.3871 (PMC10826908; doi:10.52547/ibj.3871)
Supplement: Supplementary file 1 [file ibj-27-375-s001.pdf]

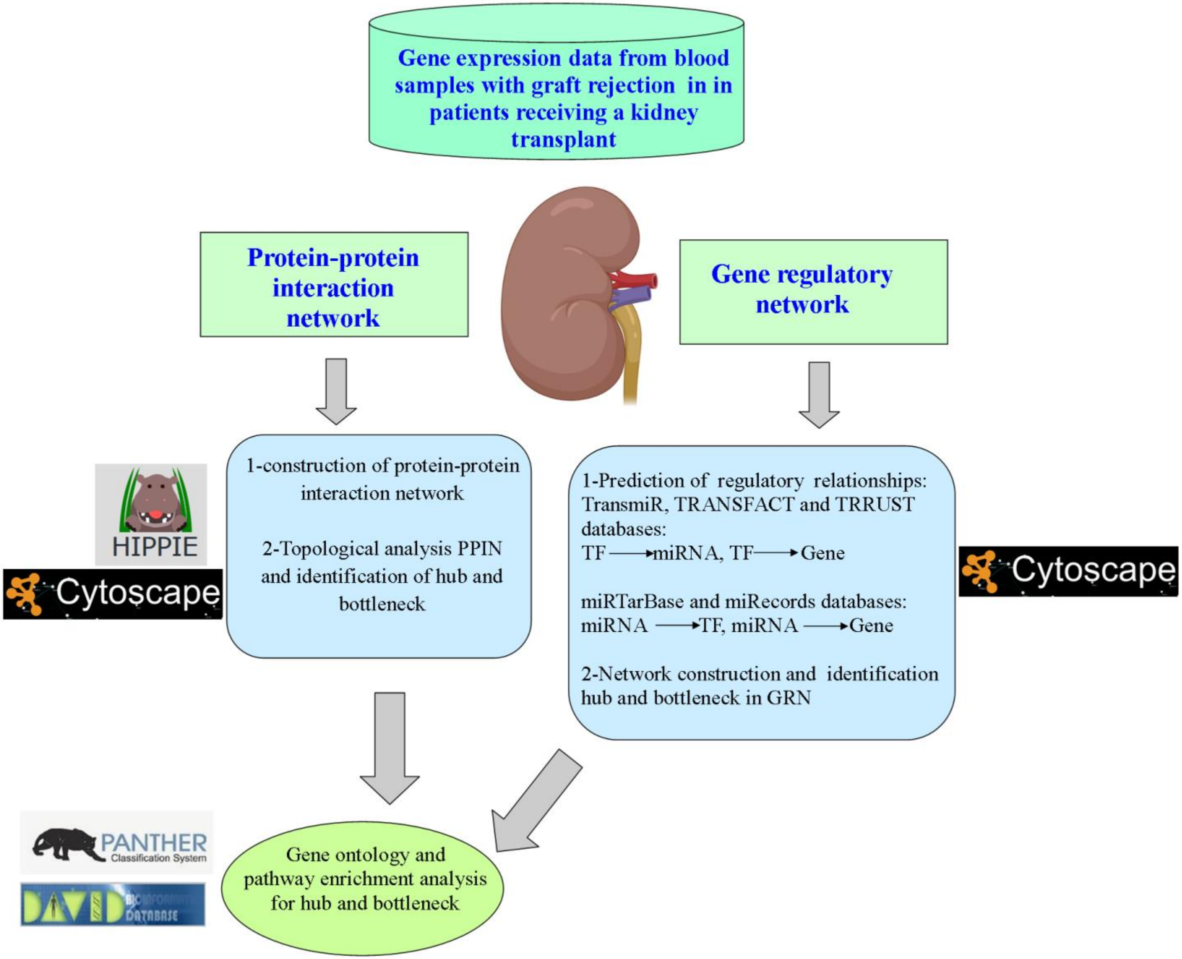

Supplementary Fig. 1. Workflow of the study.

Supplementary Table 1. Results of shared up- and downregulated genes between GSEs obtained by Venn diagram

Shared upregulated genes	Shared downregulated genes
SLC8A1-AS1	*PRKRA*
H3F3A	*SIGLEC17P*
SQSTM1	*PTGDR*
DMXL2	*ZBTB38*
NLN	*HNRNPR*
MKNK1	*CAMK2D*
CDK13	*RICTOR*
N4BP2L2	*DAPP1*
C9orf84	*ARL4C*
GBP1	*PSMF1*
PGGHG	*GUCY1B3*
DLGAP4	*PITHD1*
ZNF721	*PTGS2*
BRD8	*TOX*
HP	*LTN1*
LOC100289333	*KLF12*
CDC42SE1	*PRF1*
LYST	*STYX*
LMO2	*MARCH*
UPP1	*MFAP3L*
VPS9D1	*JAZF1*
MALAT1	*FAM104A*
IRAK3	*KLRF1*
LINC01000	*ND2*
C1QA	*RORA*
CEACAM1	*SSBP3*
ARHGAP26	*FBXO9*
WSB1	*CLINT1*
BCAT1	*DCUN1D1*
DOCK4	*TPGS2*
MCMBP	*SYNCRIP*
TMEFF2	*CPA3*
ALPK1	*KLRC2///KLRC1*
LOC101928673	*SYTL2*
SLC11A1	*FGFR1OP2*
LRRFIP1	*P2RY10*
GRAMD1A	*HTATSF1*
LOC101060275///NPIPA5///NPIPB5/// NPIPB11///LOC613037///NPIPB4///NPIPB3	*SLC25A36*
	FGFBP2
	TGFBR3
	JAK1
	CXCL5
	FAXDC2
	KLRC4-KLRK1///KLRK1
	NOV
	RAD21
	VPS13C
	USP28
	PXK
	IL2RB
	XPO7
	TMEM106B
	KLRD1
	ACTR2
	HOPX
	ZNF652

Supplementary Table 2. Results of topological analysis of PPIN and shared genes

10% degree	10% betweenness centrality	10% closeness centrality	Shared genes (10% degree-10% betweenness centrality-10% closeness centrality
SYNCRIP	*GRAMD1A*	*GRAMD1A*	*MTOR*
SQSTM1	*FAM104A*	*FAM104A*	*ARRB2*
ACTR2	*ND2*	*ND2*	*HNRNPR*
JAK1	*TPGS2*	*TPGS2*	*MAPK14*
H3F3A, H3F3B	*SYNCRIP*	*CDC42SE1*	*VCAM1*
RICTOR	*SQSTM1*	*CDC42*	*TP53*
CLINT1	*ACTR2*	*KLF12*	*PRKRA*
FGFR1OP2	*RICTOR*	*CTBP1*	*INSR*
RAD21	*JAK1*	*SLC25A36*	*ND2*
LTN1	*CLINT1*	*PTN*	*IKBKG*
CDK13	*LTN1*	*PITHD1*	*SYNCRIP*
HNRNPR	*H3F3A, H3F3B*	*FNTB*	*RICTOR*
PSMF1	*FGFR1OP2*	*PGGHG*	*CAMK2D*
LMO2	*SUMO2*	*PAXIP1*	*PRKCZ*
MCMBP	*HNRNPR*	*VCP*	*USP28*
BRD8	*CDK13*	*SRC*	*MARCH*
MKNK1	*MTOR*	*UBXN2B*	*KEAP1*
PTGS2	*RAD21*	*EWSR1*	*ICE2*
PRKRA	*LMO2*	*TTLL1*	*JAK1*
DCUN1D1	*MKNK1*	*C11orf49*	*SUMO2*
CAMK2D	*TIRAP*	*UBXN6*	*SQSTM1*
ZBTB38	*BRD8*	*NDUFA11*	*FAM104A*
XPO7	*CAMK2D*	*TIMMDC1*	*BRD8*
HTATSF1	*SNW1*	*GABRE*	*PTPN11*
TGFBR3	*IKBKG*	*MCOLN3*	*EGLN3*
MARCH	*PSMF1*	*ACVR1*	*MAPT*
NLN	*MAPK14*	*GRAMD2B*	*TARDBP*
USP28	*MCMBP*	*TMEM30A*	*GRAMD1A*
SSBP3	*UBE2D2*	*SYNCRIP*	*SNW1*
C1QA	*ICE2*	*SQSTM1*	*RIPK4*
CEACAM1	*MAPT*	*MTOR*	*CDK13*
LOC100290651, STYX	*TOP1*	*HNRNPR*	*ACTR2*
DLGAP4	*TARDBP*	*TARDBP*	*TPGS2*
HP	*PHC2*	*MAPT*	*GRB2*
SUMO2	*SEM1*	*SUMO2*	
RORA	*DCUN1D1*	*RICTOR*	
ARHGAP26	*XPO7*	*TP53*	
DMXL2	*PTGS2*	*SNW1*	
WSB1	*PRKRA*	*PRKRA*	
GRAMD1A	*KEAP1*	*JAK1*	
FBXO9	*TP53*	*PTPN11*	
SYTL2	*ARRB2*	*GRB2*	
TP53	*ZBTB38*	*TNFRSF1A*	
LRRFIP1	*STK24*	*VCAM1*	
IRAK3	*HTATSF1*	*KEAP1*	
IKBKG	*PARD6B*	*CDK13*	
DOCK4	*DYNLL1*	*IKBKG*	
N4BP2L2	*MARCH*	*MAPK14*	
VPS13C	*INSR*	*CTTN*	
SNW1	*TGFBR3*	*ECHS1*	
PXK	*PTPN11*	*CAMK2D*	
ARRB2	*MBOAT1*	*INSR*	
CUL1	*GRB2*	*ICE2*	
SKP1	*EHMT2*	*ARRB2*	
MAPK14	*NLN*	*PRKCZ*	
KEAP1	*CLTC*	*RIPK4*	
MTOR	*USP28*	*STK24*	
PTPN11	*VCAM1*	*CDK2*	
GRB2	*TGFBR2*	*ACTR2*	
EP300	*ARRB1*	*BRD8*	
HNRNPA1	*PRKCZ*	*MYC*	
TGFBR2	*PPP2CA*	*EGLN3*	
EGLN3	*DLGAP4*	*ILF2*	
JAZF1	*TNFRSF1A*	*RPTOR*	
FAM104A	*EGLN3*	*MAP1LC3A*	
VCAM1	*LOC100290651, STYX*	*MAP1LC3B*	
MAPT	*CDK2*	*GABARAPL1*	

10% degree	10% betweenness centrality	10% closeness centrality	Shared genes (10% degree-10% betweenness centrality-10% closeness centrality
TARDBP	*RXRA*	*USP28*	
ND2	*MAP1LC3A*	*HNRNPA1*	
INSR	*MAP1LC3B*	*RXRA*	
MYC	*GABARAPL1*	*CDC37*	
TIRAP	*RIPK4*	*PRMT1*	
ILF2	*C1QA*	*SMN1, SMN2*	
ICE2	*STAT1*	*YBX1*	
HOPX	*HP*	*HNRNPU*	
TPGS2	*SRPK1*	*LARP7*	
PARD6B	*CTTN*	*MEPCE*	
DBN1	*FBXW7*	*H2AFX*	
RIPK4	*CCT3*	*SNRNP70*	
CDC5L	*ECHS1*	*CNBP*	
IQGAP1	*FN1*	*U2AF2*	
CUL3	*CDC5L*	*CDK9*	
PRKCZ	*SKP1*	*PHC2*	
CUL5	*SSBP3*	*MARCH*	
MAPK1	*ARHGAP26*	*DICER1*	

Supplementary Table 3. Results of topological analysis of upregulated genes in GRN and shared genes

5% degree	5% betweenness centrality	5% closeness centrality	Shared genes (5% degree-5% betweenness centrality-5% closeness centrality
MALAT1	*MALAT1*	*MALAT1*	*VDR*
NFIC	*TP53*	*TP53*	*miR-204*
GATA6	*POU2F1*	*POU2F1*	*TP53*
TP53	*NFIC*	*miR-204*	*CRTC3*
YY1	*miR-204*	*miR-200a*	*RARA*
SP1	*NR3C1*	*miR-200b*	*LYST*
ZBTB7A	*FOXC1*	*NR3C1*	*PCBP1*
REL	*GATA6*	*CEBPB*	*miR-200a*
POU2F1	*BCAT1*	*BCAT1*	*NFIA*
HIF1A	*miR-9*	*miR-200c*	*CACYBP*
STAT3	*miR-200b*	*miR-141*	*GATA2*
WSB1	*YY1*	*AHR*	*UPP1*
CREB1	*SP1*	*SMAD3*	*SQSTM1*
KLF13	*AHR*	*THRA*	*miR-9*
FOXC1	*miR-200a*	*miR-9*	*SMAD3*
SMAD4	*GATA2*	*SREBF1*	*MALAT1*
ELK4	*THRA*	*EZH2*	*EZH2*
NR3C1	*SQSTM1*	*SQSTM1*	*miR-146b*
E2F1	*REL*	*TGFB1*	*miR-429*
ESR1	*CEBPB*	*VDR*	*miR-200b*
SRF	*SREBF1*	*PPARG*	*FOXC1*
SPIB	*VDR*	*LRRFIP1*	*SREBF1*
MAPK14	*miR-200c*	*NFIA*	*POU2F1*
ZFHX3	*miR-202*	*miR-429*	*BCAT1*
RELA	*miR-141*	*PRDM1*	*MEF2A*
PURA	*WSB1*	*TCF4*	*TGFB1*
RUNX1	*LRRFIP1*	*RARA*	*CEBPB*
UBTF	*HIF1A*	*miR-146b*	*SND1*
ZNF281	*miR-146b*	*miR-22*	*TMEFF2*
E2F6	*ZBTB7A*	*MEF2A*	*ETS2*
SP2	*UPP1*	*FOXC1*	*miR-200c*
BCAT1	*FOXC1*	*miR-202*	*PPARG*
AHR	*STAT3*	*ETS2*	*miR-22*
WT1	*miR-21*	*SND1*	*LRRFIP1*
THRA	*KLF13*	*CRTC3*	*TCF4*
TFAP2A	*ZNF281*	*PCBP1*	*PRDM1*
NFKB1	*MAPK14*	*miR-375*	*AHR*
ETS1	*SRF*	*GATA2*	*miR-141*
REPIN1	*RARA*	*TMEFF2*	*NR3C1*
CEBPB	*RELA*	*BCL6*	*THRA*
NLN	*PCBP1*	*LYST*	
MYC	*ELK4*	*DNMT1*	
miR-155	*CREB1*	*HDAC2*	
SQSTM1	*miR-22*	*CACYBP*	
HNF4A	*E2F6*	*E2F7*	
IRAK3	*NLN*	*SP3*	
ELK1	*miR-429*	*ATF4*	
miR-21	*CDC42SE1*	*IL6*	
VDR	*miR-155*	*PPARA*	
DLGAP4	*THRB*	*BMP4*	
JUN	*ETS2*	*BMI1*	
CDC42SE1	*CRTC3*	*AKT1*	
FOS	*RUNX1*	*KLF3*	
miR-17	*ELK1*	*NFATC3*	
miR-20a	*SMAD4*	*EP300*	
SREBF1	*REPIN1*	*IRF3*	
LRRFIP1	*ZFHX3*	*IL1B*	
N4BP2L2	*miR-23c*	*lincMTX2*	
miR-200c	*TGFB1*	*UPP1*	
APEX1	*SMAD3*	*TEAD2*	
ZNF148	*NFIA*	*CDKN1A*	
THRB	*SPIB*	*NR2F1*	
SNAI2	*SND1*	*BRD4*	
miR-200b	*E2F1*	*JMJD1C*	
NR1H2	*ETS1*	*SMAD1*	
miR-19a	*IRAK3*	*EMSY*	

5% degree	5% betweenness centrality	5% closeness centrality	Shared genes (5% degree-5% betweenness centrality-5% closeness centrality
POU2F2	*PRDM1*	*FOXM1*	
MTF1	*lincMTX2*	*PELP1*	
EGR1	*TMEFF2*	*AKT2*	
H3F3A	*EZH2*	*EHMT2*	
miR-145	*miR-20a*	*SIX1*	
RARA	*miR-17*	*miR-23c*	
ZBTB16	*HNF4A*	*CDKN2A*	
miR-27a	*miR-375*	*lincFOXF1*	
miR-200a	*ESR1*	*PBX2*	
TCF3	*miR-93*	*HEY1*	
miR-34a	*JUN*	*ITGB3*	
miR-18a	*NFKB1*	*PLK1*	
miR-93	*miR-145*	*miR-1297*	
SREBF2	*UBTF*	*PCGF2*	
HINFP	*ZNF148*	*DDIT3*	
miR-141	*TCF4*	*GAS5*	
UPP1	*CDK13*	*MTA1*	
MYCN	*PPARG*	*BCLAF1*	
KLF4	*CACYBP*	*HDAC9*	
SOX9	*FOS*	*ATF3*	
miR-146a	*H3F3A*	*HPS1*	
CDK13	*miR-122*	*LOC100133286*	
NFYA	*miR-124*	*LOC100132942*	
miR-143	*PURA*	*LOC100132834*	
miR-124	*DLGAP4*	*LOC100132474*	
miR-106b	*MTF1*	*NBPF10*	
MCMBP	*miR-143*	*GPS2P2*	
miR-429	*LMO2*	*LOC100131910*	
FOXL1	*LYST*	*LOC100131821*	
HOXA5	*ARHGAP26*	*LOC100131540*	
SAMD9L	*miR-223*	*LOC100131195*	
GRAMD1A	*WT1*	*LOC100131150*	
PCBP1	*TFAP2A*	*LOC100131053*	
SNAI1	*MCMBP*	*LOC100130876*	
IRF1	*MEF2A*	*LOC100130856*	
miR-221	*N4BP2L2*	*LOC100130542*	
LMO2	*miR-31*	*LOC100130468*	
MYB	*lincFOXF1*	*LOC100129974*	
RUNX2	*miR-146a*	*NAG20*	
HOXD9	*POU2F2*	*LOC100129380*	
ARHGAP26	*CEBPD*	*LOC100129363*	
ETS2	*miR-221*	*LOC100129322*	
FOXA1	*miR-27a*	*LOC100129312*	
BRCA1	*miR-18a*	*RPL22P24*	
SPI1	*miR-29a*	*LOC100129280*	
miR-20b	*miR-34a*	*LOC100129233*	
RXRA	*GRAMD1A*	*LOC100129171*	
miR-150	*SP2*	*LOC100129118*	
miR-106a	*miR-106b*	*LOC100129069*	
miR-182	*TCF3*	*LOC100129042*	
miR-29a	*BCL6*	*LOC100128343*	
PRDM1	*miR-19a*	*LOC100128325*	
SND1	*let-7b*	*LOC100128088*	
IRF2	*RAD51-AS1*	*LOC100125918*	
miR-92a	*GAS5*	*LOC730139*	
SLC11A1	*CEACAM1*	*MOSMO*	
miR-16	*miR-142*	*LOC729696*	
CEACAM1	*MYC*	*ZNF812P*	
CEBPD	*miR-222*	*ANKDD1B*	
EZH2	*miR-1*	*LOC728543*	
NFIA	*miR-16*	*SNORD89*	
TGFB1	*SLC11A1*	*SCARNA10*	
CRTC3	*NORAD*	*SNORD23*	
CBFB	*ZBTB16*	*SNORD6*	
miR-23a	*SNAI2*	*SNORA16A*	
let-7b	*HP*	*SNORD5*	
miR-31	*SAMD9L*	*SNORA32*	
TMEFF2	*APEX1*	*SNORA25*	

5% degree	5% betweenness centrality	5% closeness centrality	Shared genes (5% degree-5% betweenness centrality-5% closeness centrality
SMAD3	*GBP1*	*SNORA61*	
miR-19b	*miR-192*	*SNORA44*	
TP63	*DNMT1*	*SNORA80E*	
let-7a	*RXRA*	*SNORA40*	
miR-15a	*miR-182*	*SNORA2C*	
miR-1	*miR-15a*	*SNORA30*	
miR-223	*ATF4*	*SNORA28*	
miR-92a-1	*miR-214*	*SNORA18*	
JUND	*let-7a*	*SNORA2A*	
miR-183	*CRTC1*	*SNORA1*	
miR-302b	*lnrCXCR4*	*SCARNA23*	
miR-96	*miR-20b*	*SCARNA4*	
CRTC1	*miR-519d*	*SNORA13*	
miR-222	*miR-96*	*SNORA8*	
miR-9	*miR-215*	*TMEM183B*	
miR-19b-1	*HDAC2*	*HIST2H3D*	
STAT1	*DOCK4*	*PHLDB3*	
miR-27b	*miR-26b*	*ABCA17P*	
DOCK4	*DMXL2*	*LOC648570*	
CRX	*HOXA5*	*LOC646870*	
GATA2	*miR-106a*	*LOC646644*	
ARNT	*ALPK1*	*LOC646517*	
miR-519d	*ARNT*	*LOC645937*	
miR-192	*MKNK1*	*MAGEB17*	
miR-122	*miR-424*	*LOC643733*	
STAT5B	*miR-125a*	*CCER2*	
SOX2	*miR-149*	*SNORA4*	
TFAP2C	*miR-7*	*LOC606724*	
miR-302d	*TP63*	*BOLA2*	
miR-205	*SPI1*	*DNAJC25*	
miR-373	*NR1H2*	*LRRC37A2*	
DMXL2	*ELF3*	*OR9K2*	
miR-29b	*NFYA*	*RP9P*	
BRD8	*EGR1*	*PMS2CL*	
miR-25	*IL6*	*MTHFD2L*	
miR-125a	*C1QA*	*LOC440895*	
miR-424	*miR-150*	*LINC01123*	
miR-26b	*HINFP*	*HIST2H2BF*	
miR-22	*E2F7*	*SMG1P2*	
miR-146b	*let-7c*	*EIF2AK4*	
FOXC1	*PBX2*	*C7orf61*	
miR-204	*miR-373*	*XKRX*	
NFE2L2	*miR-98*	*SRRD*	
miR-23b	*KLF4*	*LOC401317*	
miR-130b	*miR-302b*	*FLJ46552*	
C9ORF84	*miR-25*	*NBPF9*	
let-7c	*miR-19b*	*ZNF772*	
miR-7	*miR-101*	*LOC400682*	
MKNK1	*miR-449a*	*C18orf49*	
miR-214	*miR-144*	*MED11*	
miR-142	*miR-193b*	*ST20*	
miR-130a	*miR-181a*	*SHC4*	
ZNF721	*miR-372*	*FAM102A*	
miR-15b	*miR-15b*	*ZNF805*	
miR-372	*miR-23a*	*OR51I2*	
miR-181a	*miR-205*	*PPIAP19*	
miR-101	*miR-92a*	*FAM180A*	
miR-149	*DA125942*	*TEX43*	
TCF4	*C9ORF84*	*C1orf189*	
E2F3	*miR-185*	*GBP7*	
TWIST1	*miR-27b*	*ZNF470*	
miR-302a	*NFATC3*	*ZNF790*	
miR-302c	*miR-190a*	*YPEL2*	
miR-30b	*miR-506*	*FIBIN*	
miR-30a	*BRD8*	*SLC16A12*	
miR-30d	*miR-302d*	*AMIGO3*	
miR-29c	*BRCA1*	*RNF138P1*	
miR-186	*SP3*	*CA13*	

5% degree	5% betweenness centrality	5% closeness centrality	Shared genes (5% degree-5% betweenness centrality-5% closeness centrality
miR-125b	*miR-125b*	*RAB15*	
miR-215	*HIPSTR*	*C5orf34*	
LYST	*HPSE*	*C3orf62*	
GATA2	*miR-186*	*RBM43*	
ZEB1	*miR-6778*	*MIGA1*	
AR	*FOXF2*	*SVBP*	
miR-24-2	*CREM*	*ZNF773*	
miR-218	*HOXD9*	*ZNF568*	
FOXF2	*miR-137*	*SBSN*	
ELF3	*STAT1*	*ZNF699*	
PPARG	*miR-132*	*LRRC37A3*	
MITF	*FOXA1*	*FAM111B*	
LTF	*miR-211*	*NDUFS7*	
miR-320a	*RUNX2*	*CDRT1*	
miR-24	*miR-4755*	*USP50*	
CREM	*miR-218*	*DPPA3*	
miR-190a	*IRF1*	*BMP8A*	
miR-193b	*miR-29c*	*ZNF445*	
C1QA	*miR-135b*	*ZNF429*	
ALPK1	*miR-24*	*EP400P1*	
CACYBP	*TFAP2C*	*AMIGO2*	
MAX	*miR-423*	*KIF24*	
miR-193a	*SOX2*	*SLC10A5*	
miR-185	*IRF2*	*FAM174A*	
DA125942	*miR-30d*	*LRIT3*	
GBP1	*MYCN*	*ZNF774*	
HP	*miR-337*	*OVCH1*	
STAT5	*FOXL1*	*ZDHHC21*	
miR-206	*CDKN1A*	*NRBP2*	
miR-203a	*CAT2*	*TMEM173*	
miR-195	*miR-130b*	*C3orf35*	
let-7e	*ZNF721*	*LOC339803*	
miR-107	*miR-4768*	*SPOPL*	
miR-8485	*lincIRX5*	*ZNF260*	
PITX1	*miR-342*	*METTL2A*	
miR-34c	*miR-183*	*RAB43*	
miR-5011	*miR-503*	*CHSY3*	
miR-4755	*miR-32*	*HIST2H2AB*	
miR-211	*miR-140*	*TRIM59*	
miR-98	*miR-4722*	*ATP11C*	
MEF2A	*miR-30a*	*FOXD4L3*	
CEBPA	*miR-615*	*C9orf47*	
miR-29b-1	*miR-320a*	*LOC286161*	

Supplementary Table 4. Results of topological analysis of downregulated genes in GRN and shared genes

5% degree	5% betweenness centrality	5% closeness centrality	Shared genes (5% degree-5%betweenness centrality-5% closeness centrality)
NFIC	*NFIC*	*miR-17*	*miR-21*
GATA6	*GATA6*	*miR-20a*	*YY1*
TP53	*TP53*	*miR-93*	*FOXC1*
MYC	*ZNF652*	*miR-155*	*FOS*
YY1	*YY1*	*miR-18a*	*KLF13*
ZNF652	*RORA*	*miR-106b*	*miR-92a*
SP1	*SP1*	*miR-124*	*miR-20a*
HMGA1	*MYC*	*miR-106a*	*miR-155*
ZBTB7A	*ZBTB7A*	*miR-519d*	*SPIB*
RORA	*HMGA1*	*MYC*	*UBTF*
REL	*REL*	*miR-20b*	*miR-145*
POU2F1	*SYNCRIP*	*miR-16*	*MAPK14*
HIF1A	*POU2F1*	*TP53*	*TP53*
STAT3	*HIF1A*	*SP1*	*miR-34a*
SYNCRIP	*PTGS2*	*NFIC*	*REL*
CREB1	*miR-155*	*let-7b*	*FOXO3*
ACTR2	*STAT3*	*miR-186*	*miR-19a*
KLF13	*KLF13*	*GATA6*	*miR-93*
FOXC1	*ACTR2*	*miR-101*	*EP300*
SMAD4	*ELK4*	*miR-19a*	*POU2F1*
ELK4	*miR-21*	*miR-98*	*SMAD4*
E2F1	*FOXC1*	*STAT3*	*SYNCRIP*
NR3C1	*ARL4C*	*ZNF652*	*TGFBR3*
ESR1	*TGFBR3*	*YY1*	*CEBPB*
SRF	*KLRD1*	*HIF1A*	*RICTOR*
TGFBR3	*NR3C1*	*miR-21*	*HMGA1*
SPIB	*SLC25A36*	*REL*	*NFIC*
MAPK14	*CREB1*	*RORA*	*MYC*
RELA	*RELA*	*miR-15a*	*miR-124*
ZFHX3	*MAPK14*	*RELA*	*JUN*
PURA	*SMAD4*	*miR-92a*	*USP28*
ARL4C	*miR-17*	*POU2F1*	*E2F1*
SLC25A36	*REPIN1*	*CREB1*	*RORA*
RUNX1	*SRF*	*ZBTB7A*	*TCF7L2*
KLRD1	*ZNF281*	*HMGA1*	*CXCL5*
UBTF	*miR-20a*	*miR-526b*	*miR-18a*
TMEM106B	*TMEM106B*	*SMAD4*	*ETS1*
ZNF281	*RBPJ*	*miR-190a*	*ARL4C*
MYCN	*SPIB*	*miR-15b*	*MYCN*
RBPJ	*ZFHX3*	*ACTR2*	*STAT3*
miR-155	*PXK*	*ETS1*	*HNF4A*
E2F6	*MYCN*	*E2F1*	*RELA*
STYX	*miR-93*	*TGFBR3*	*HIF1A*
CXCL5	*ELK1*	*miR-192*	*FAM104A*
TCF7L2	*E2F6*	*NFKB1*	*ZFHX3*
AHR	*STYX*	*miR-5011*	*SRF*
ATM	*E2F1*	*let-7a*	*miR-106b*
PTGS2	*CXCL5*	*ESR1*	*TMEM106B*
WT1	*RUNX1*	*FOXO3*	*RUNX1*
FAM104A	*PURA*	*miR-8485*	*PTGS2*
TFAP2A	*UBTF*	*miR-218*	*CTNNB1*
NFKB1	*miR-124*	*miR-125a*	*miR-221*
ETS1	*ESR1*	*miR-221*	*miR-17*
REPIN1	*TCF7L2*	*miR-183*	*NFKB1*
miR-21	*ETS1*	*miR-25*	*ELK4*
FOXO3	*miR-200c*	*SRF*	*SP1*
ZBTB38	*TFAP2A*	*SYNCRIP*	*ZBTB38*
CEBPB	*ZBTB38*	*NR3C1*	*AHR*
miR-17	*miR-18a*	*miR-145*	*SLC25A36*
miR-20a	*let-7b*	*ELK4*	*RBPJ*
ELK1	*miR-145*	*RBPJ*	*ACTR2*
HNF4A	*FOXC1*	*MAPK14*	*ZNF652*

5% degree	5% betweenness centrality	5% closeness centrality	Shared genes (5% degree-5%betweenness centrality-5% closeness centrality)
RICTOR	*RICTOR*	*miR-26b*	*GATA6*
JUN	*USP28*	*FOXC1*	*miR-27a*
STAT2	*THRB*	*SPIB*	*NR3C1*
HNRNPR	*HNRNPR*	*MYCN*	*ZBTB7A*
FOS	*ATM*	*RUNX1*	*PURA*
PXK	*miR-221*	*PURA*	*ATM*
MAX	*miR-122*	*miR-373*	*ESR1*
USP28	*FAM104A*	*miR-34a*	*CREB1*
miR-200c	*FOXO3*	*TCF7L2*	
SREBF1	*FOS*	*JUN*	
CTNNB1	*WT1*	*TMEM106B*	
EP300	*miR-141*	*CEBPB*	
PPARA	*miR-106b*	*miR-27a*	
miR-19a	*CEBPB*	*miR-1277*	
APEX1	*AHR*	*EP300*	
FBXO9	*miR-101*	*PPARA*	
miR-93	*miR-143*	*KLF4*	
TEAD1	*HNF4A*	*miR-548ah*	
PSMF1	*JUN*	*miR-215*	
miR-18a	*miR-16*	*miR-195*	
ZNF148	*MTF1*	*miR-143*	
THRB	*miR-26b*	*miR-497*	
SYTL2	*miR-27a*	*RICTOR*	
miR-200b	*NFKB1*	*FOS*	
miR-34a	*FBXO9*	*SLC25A36*	
miR-27a	*STAT2*	*FOXC1*	
KLF12	*miR-223*	*miR-185*	
SNAI2	*PSMF1*	*FAM104A*	
FGFR1OP2	*MAX*	*CTNNB1*	
NR1H2	*miR-19a*	*ARL4C*	
MTF1	*miR-31*	*miR-19b*	
POU2F2	*miR-9*	*miR-363*	
miR-200a	*EP300*	*HNF4A*	
miR-145	*miR-106a*	*miR-31*	
CREBBP	*ZNF148*	*PTGS2*	
P2RY10	*CTNNB1*	*ZBTB38*	
miR-124	*RAD21*	*miR-204*	
EGR1	*miR-186*	*miR-32*	
miR-141	*FGFR1OP2*	*miR-372*	
ZBTB16	*CREBBP*	*miR-130b*	
RARA	*miR-29a*	*SNAI2*	
TCF3	*miR-146a*	*USP28*	
miR-106b	*miR-34a*	*AHR*	
SREBF2	*KLF12*	*miR-128*	
RAD21	*miR-92a*	*CXCL5*	
HINFP	*PCBP1*	*SMAD3*	
miR-221	*miR-20b*	*ZFHX3*	
JAZF1	*miR-98*	*KLF13*	
HOPX	*miR-519d*	*miR-223*	
IL2RB	*let-7a*	*ATM*	
miR-92a	*HOPX*	*UBTF*	
